# The Characteristics of Ten Tissue Remedies

**Published:** 1888-04

**Authors:** E. J. Lee


### THE CHARACTERISTICS OF TEN TISSUE REMEDIES.

Most of the ten remedies to which I call your attention are now prescribed chiefly upon clinical indications. It shall be my endeavor to bring forward some few symptoms, which may guide us in our use of these drugs. They have, many of them at least, been neglected in our study and use of the materia medica.

### CALCAREA FLUORICA.

For our knowledge of the pathogenesis of this remedy, we are chiefly indebted to Dr. J. B. Bell, of Boston. The drug, as proven by him and his associates, shows symptoms which give promise of usefulness when further proven and studied.

Mentally, we find depression of spirits, with a marked fear of financial ruin. (Note.—Other drugs have a similar symptom; thus, patients think they are poor, is found under Bell., Hepar, Nux-v., *Sepia*, Valer. The Arsenic patient thinks his family will starve.) So far clinical experience has shown Calc-fl. to be useful in cases of indurated glands (compare with Asterias, Baryta, Conium, Phytolacca, Silicea, Sulphur, etc.), like the mammæ or testicles; also for nodes and exudations on bones—these latter are usually irregular in shape—and for suppurations of bony tissues. (Calc-p., Sil., etc.)

The pains are generally worse in damp weather but are ameliorated by fomentations; are also better from lying on the painless side and from motion. There is a backache, very like that of Rhus, caused by a strain, worse from rest, better from motion and warmth. We also note a cough from tickling in the larynx, not relieved by coughing (which is very like the cough of Ignatia and Marum). The urine is more profuse, especially at night; the patient is awakened at night by an itching at the anus from pin worms. The only peculiarities we observe under this drug are the fear of financial ruin, the stony hardness of its glandular swellings, and the irregular shape of its bony growths. The mental symptom is also found under other drugs which are frequently indicated in diseases of bones and glands.

### CALCAREA PHOSPHORICA.

This drug is so well known that I shall pass it over with a very brief notice, it being our intention this evening to study those remedies which have not been so thoroughly proven or so much used.

The mental condition of Calc-ph. is dull, obtuse ; also anxiety. Patient is worse from grief or bad news and when thinking of his complaints. Like Agar., Bar., Chin., Ferr., Helon., Stram., Thuja, the Calc-ph. patient is better when occupied. The aggravations from thinking of ailments and from exposure to cold, damp weather are the prominent conditions of this remedy. Vertigo in old, debilitated persons. (Also Baryta, Carbo-v.)

The headache is generally of a dull kind, which is better from cold applications and when occupied mentally. Smoking also relieves the headache. In the headaches of school girls we think of Calc-ph. or of Phos-ac. With Calc-ph. these girls show signs of mal-nutrition ; with Phos-ac. the headache is rather from overstudy. (We must also recollect the Silicea pains, etc., in growing children.) With thin, emaciated, peevish babies, whose fontanelles remain open too long, or close and open again, we think of this remedy. (Here, as elsewhere, it is important to distinguish between Calcarea c. and Calc-ph. Both have the large head and the open fontanelles; Calc-c. has enlarged, swollen abdomen, the stools are generally white, there is a craving for eggs, profuse sweat on back of head during sleep ; it has not the aggravation from thinking of complaints, nor so marked an aggravation from damp weather.) Calc-ph. is very useful in the tardy dentition of thin, fretful children, whose bones do not properly develop. Infants, who want to nurse all the time and yet don't thrive. They will vomit their food continually, whether it be mother's milk or other food. They have colic after each feeding. Sometimes they will refuse to take the breast because the milk is too salty.

There is a coryza which is fluent in a cold room, but stopped up in warm air or out-doors, with an increased flow of saliva. The face is pale and thin yet the head is large. Fauces are swollen ; warm drinks do not hurt; swallowing the saliva pains more than food. (Also Cocc., *Lach.*, Merc., Crotal.) Pain in the throat, chest, and epigastrium on swallowing.

The appetite comes when thinking of food. (The reverse of Mur-ac., Sars., etc.) Sometimes there is a want of appetite before or during menses. A craving for ham or for smoked meats. Nausea and pressure in stomach, which are relieved when at rest. Pain in the stomach from least morsel of food. Oozing of blood from the navel of infants. (Also Abrotanum, which remedy it resembles in marasmus.) The pains in the abdomen are better after stool, by passing of flatus and after leucorrhœa. The diarrhœa is generally greenish, hot, and often profuse and watery.

Calc-ph. has nymphomania; aching, pressing, or weakness in uterine region; prolapsus in debilitated persons, etc. All of its uterine symptoms are to be found in women who are debilitated or who have rheumatic pains from the least exposure to cold, damp weather. (Wherein it strongly resembles Dulcamara.) (In its various menstrual or leucorrhœal troubles in rheumatic females, Calc-ph. resembles Cimic. and Caul.)

Chest troubles in persons suffering from fistula in ano, especially when the symptoms seem to alternate between the chest and the anal trouble. (Berberis also.)

We find rheumatic pains in back and limbs, worse from motion and from least exposure to dampness. Calc-ph. is especially indicated in women whose joints ache and pain at every change of the weather. When after exposure to dampness we find stiffness of neck with aching and soreness of limbs; wandering pains in the limbs, especially in sacral region and down the legs; all worse at any change of the weather. Rheumatism with uterine displacements.

Calc-ph., with Symphytum, is often indicated in cases where the bones do not grow together after a fracture.

Hering tells us to compare Calc-ph. with Berb., *Calc-c.*, Calc-fl., *Fluor-ac.*, Ruta, *Silicea*, and Sulphur, in bone affections, fistulæ, etc.

In joint affections, rheumatic and suppurative, with Berb., Kali-phos., and Natr-mur.

In dental caries, with Fluor-ac., Mag-phos., and Silicea.

In epilepsy, with Calc., Ferr-ph., Kali-mur., Kali-ph., and Silicea.

In spasms of the eyelids, with Calc. and Nux-v.

In diabetes, with Kali-ph., Nat-ph.

In tabes mesenterica, with Ars., Iodum, and Merc.

In hemorrhages, with Ferr-ph.

In worm affections, with Nat-ph. (Removes disposition.)

In debility after acute diseases, with Psorinum, which has greater tendency to profuse sweats.

In the peevishness of children, with Cham., Cina, and Kali-ph.

### CALCAREA SULPHURICA.

Hering tells us that "Calc-sul. resembles Hepar but acts more intensely and more deeply, and is often useful after Hepar has ceased to act." From the meagre record of this drug, given in the *Guiding Symptoms*, we find it is chiefly useful for inflammations and suppurations; for effects of blows, etc. For the eye, after an injury by a splinter, like Acon., Silicea, and Symphytum. For otitis, after a blow on the ear. (Compare with Arn.) The pus discharged is thick, yellow, as is also the nasal discharge. Of the skin symptoms, we read of "many tender pimples under the beard, exuding an oily matter." (Other remedies having these pimples are, Agar., Ambra, Calc., Graph., Lach., Nitr-ac.) With Calc-sul. cold drinks temporarily relieve the colic; washing the face with cold water relieves an excoriating coryza. There is general aggravation from working and washing in water.

### FERRUM PHOSPHORICUM.

The following indications for Ferr-ph. are given by Farrington: The pulse is full, round, and soft; the inflammation has not yet gone on to exudation; it is merely what is termed dilatation of the blood-vessels. The chest is sore and bruised. If there be a discharge of pus or muco-pus, then Ferr-ph. is not the remedy. The expectoration is blood streaked from simple congestion. If a patient with phthisis take cold, and so become greatly prostrated and have a blood-streaked expectoration, then Ferr-ph., even in the 200th potency, would quickly relieve the pulmonary congestion. So, too, in secondary inflammation following pneumonia; one lung is inflamed, when suddenly the other becomes congested. Here, again, Ferr-ph. acts. Or, suppose, on a warm summer's day, a child is exposed while perspiring and takes cold. In consequence inflammation of the bowels sets in; the stools are watery and bloody; there may be some urging but no tenesmus. Here, too, Ferr-ph. would relieve.

Aconite has a full, bounding pulse, with dry heat of the skin, anxiety, fear of death, and restlessness. Its symptoms betoken *active* congestion, while those of Ferr-ph. indicate rather a *semi-paretic* condition of the blood-vessels. Gelsemium is more like Ferr-ph., but with its fever there are prostration, drowsiness, and paralysis of muscles; they will not obey the will. Patient is drowsy and wants to remain quiet.

The pains of Ferr-ph. are said to be aggravated by motion and relieved by cold.

### KALI MURIATICUM.

Potassium chloride has not been proven, therefore we have no reliable data for its use. Clinically or empirically, it is chiefly recommended for glandular swellings or for rheumatic, gouty pains which are worse from motion, accompanied by white or grayish coating at the base of the tongue. These indications are not to be relied on, as they are far too vague and are only clinical. Why should one use such remedies as this when he has many well-proven remedies which cover the same ground? Bryonia, for instance, has the symptoms which are given as indicative of Kali-mur.

### KALI PHOSPHORICUM.

Potassium phosphate is also an unproven drug, and hence we have no reliable data for its use. Clinically it is recommended for "neuralgic pains, occurring in any organ, with depression of spirits, failure of strength, sensitiveness to light and noise; improved by pleasant excitement and from gentle motion, but is mostly felt when quiet or alone." (Bœricke and Dewey.) These symptoms remind us of Pulsatilla.

### KALI SULPHURICUM.

Of Potassium sulphate we have practically no provings, as the scanty records given in the *Encyclopædia* are useless. Clinically it has been recommended for loose, rattling cough, which hangs on after other symptoms have been relieved, when patient is worse indoors and better out in the air.

In yellow, thick mucous discharges with these conditions it has cured. Two interesting cases are reported by Dr. Wm. P. Wesselhœft. (See Drs. Bœricke and Dewey on the *Twelve Tissue Remedies*, p. 113.) We quote from these cases the following:

1. Thick, yellow, offensive ozæna, alternating with watery discharge; has been affected with it for eighteen months; has lost taste and smell; left nostril worse. Catamenia every three weeks. Takes cold very easily. Still-born child three years ago. Kal-sul.$^{12}$, in water, to be taken once a week. In one month reported catarrh entirely well; has regained much of the lost senses of taste and smell.

2. Male, light complexion; about once a week a thick, dark brown, semi-fluid accumulation of pus formed in the left upper nostril; on being blown out, it omitted a terrible stench. About a month previous a piece of carious bone was taken from the antrum Highmori through an upper left alveolus, from which a tooth had been drawn four years previous. The probe entered the antrum freely. Calcarea, Silicea, and several other remedies

12

proved inefficacious. Three weeks after having taken two doses of Kali-sul.$^{6}$, in water, morning and evening, a tablespoonful, for four days, nothing more remained of the discharge, and the alveolus closed so that no probe entered.

Kali-sul. is also used for wandering rheumatic pains. Its characteristic conditions are like those of Pulsatilla—aggravation in the evening and in a heated room, better out in the cool air.

### MAGNESIA PHOSPHORICA.

The phosphate of Magnesia is also an unproven drug; its clinical history gives promise of a very useful remedy if well proven. Its clinical use in the past has been chiefly confined to shooting neuralgic pains, severe neuralgic pains of head, eyes, or face, which are right-sided, shooting, very severe, and are relieved by external warm applications. These seem to be the indications for Mag-ph.

It is also useful for a colic, often flatulent, which causes the patient to bend double; is better from rubbing, from warmth, and is accompanied by eructations which do not relieve. These few notes will recall to your minds the case mentioned at the last meeting of the I. H. A. by Dr. Wesselhœft. It may be well to quote it here, to further illustrate the action of this remedy. Concerning Mag-ph., Dr. Wesselhœft said: "I made a cure with Mag-ph. very similar to the one Dr. Nash has just reported. It is one, I think, could never have been made without Mag-ph.$^{cm.}$ It was an astonishing cure. The case was of a neuralgic character; the patient an old lady of sixty-six. I saw her first about six years ago. I wondered that any cure could be made on any one who was so attenuated, so thin, and so lacking in vitality. I have observed that Mag-ph. has three peculiarities—it is an entirely right-sided remedy, the pains are chiefly supra-orbital, shifting, and are relieved by warmth." (See Homœopathic Physician, Vol. VII, p. 254.) (Silicea will be remembered as having this relief from warm external applications. Cinnab. and Iris have relief from heat of the hand.)

### NATRUM PHOSPHORICUM.

Under the supervision of Dr. Farrington provings were made of Sodium phosphate; they were mostly with the high potencies. Mentally, we find chiefly anxiety and fear of evil of some kind. Awakens at night with anxiety. Melancholy after seminal emissions. Awakened at night, fears his child, who has a trifling ailment, is dead; he goes to her room to see. (You will remember that Natr-m. has a very similar feeling, in that after dreaming of robbers will not believe they are not in the house until search is made.) Is easily startled by the least noise, especially at night, causing palpitation. Headaches in afternoon, after menses. Headache better after breakfast, worse after dinner. Ears, intolerable burning and itching of right ear, has to scratch until it bleeds. Nose as if full of mucus. Great fullness at root of nose; skin feels drawn tightly over it in evening. Left nostril sore, painful; picks it continually; scabs form. Dropping of thick, yellow mucus from posterior nares, worse at night, awakens him, must sit up and clear the throat. Pricking in throat, worse swallowing liquids, better from solids. Tongue coated yellow (creamy or golden). Blisters on tip; sensation of hairs on tip, followed by prickling numbness of whole mouth. (Sensation as of hair on tongue is found under All-sat., Kali-bi., Natr-m., and Silicea.)

Stomach, canine hunger, cannot wait for dinner (also Sulphur). Gone feeling in stomach, morning on rising, or at eleven to twelve A. M. (at eleven A. M. is also under Hydr., Lach., *Sulph.*, and Zinc.); empty, gone feeling all day, but worse after eating (also Sang.); desire for beer, which relieves this gone feeling. Heaviness or pressure in stomach better after eating. Averse to bread (also Nat-m. and Sulph.) and butter, lasting for weeks.

Abdomen, while at stool, sensation as if a marble dropped in left abdomen. Pain through right groin day after an emission.

Rectum, etc. Sore, raw feeling at anus, with desire to retract the anus, which relieves. Rawness at anus, with desire to scratch it. After coitus urging to stool and to urinate. Must bring will to bear to prevent the escape of feces. [Like Aloe.] After a large, soft stool feels as though some remained behind.

Urinary, etc. Urine increased, must strain to pass, must wait before it will pass. Frequent seminal emissions, with or without dreams. Emissions after coitus. Semen thin, watery, smells like stale urine.

With the female the menses are too early, with aggravation of symptoms afterward; especially of headache, of palpitation, etc. During the menses icy-cold feet by day, burning at night, in bed. Empty feeling in chest and abdomen after a meal. In the lower extremities we have a variety of pains, chiefly felt when walking. Legs suddenly give way, as if paralyzed, when walking. Sleep is restless, especially during and after the menses. (Restless or disturbed sleep in connection with the menstrual flow is often complained of; the following remedies have this symptom during menses: Alum., Amm-c., Eupion, Gent-c., Kali-c., Mag-c., Natr-m.) The most marked condition of this remedy is the amelioration of the heaviness at epigastrium and the pressure by eating.

### NATRUM SULPHURICUM.

In speaking of the sulphate of Sodium I shall quote largely from the lecture on "Natrum Sulphuricum and Sycosis," by Dr. Kent, published in THE HOMŒOPATHIC PHYSICIAN, Vol. VI, page 275. This is perhaps the most valuable of all the drugs to which your attention has been called this evening; it deserves more study than has been given to it.

Mentally we have anxiety, irritability, desire for death, aversion to life and the things that generally make life pleasant and agreeable. So great is this satiety of life the patient has to *use restraint* to prevent doing herself harm; this is very characteristic of Natr-sul. Satiety of life, aversion to life, great sadness, great despondency, with irritability and dread of music—music makes her weep, makes her sad or melancholy. These symptoms run through all the Natrums, but are especially strong under Natr-sul. Cheerful or happy *after stool*, which is so characteristic of Borax, is also found under Natr-sul.

Under the head we have violent crushing pains, especially at the base of the brain. Sick headache with bilious stools or vomit. In chronic cases of conjunctivitis with granular lids, green pus, extreme photophobia, so great that patient can't open his eyelids, lest the light of the room bring on headache and great distress—these, together with the aversion to life, strongly indicate Natr-sul. (It is here to be compared with Graphites.) Earaches, worse on going from cold air into warm room, in damp weather or after living on damp ground. Stopped, stuffed-up nose; nose-bleed during the menses. (Natr-ars. has this stopped nose more strongly than any of the Natrum compounds. The patient feels stuffed up in nose and chest; nose stopped at night, must breathe through mouth; nasal discharge is yellow; mucus drops from posterior nares into throat [also Natr-ph.]; pieces of hardened bluish mucus blown from nose.)

There is a toothache worse from warm drinks and intolerably aggravated by hot ones; lessened in cool air. (Aggravation from *hot* food, Bell., *Calc.*, Ph-ac.; Bryonia is worse from warm and better from cold food. Amelioration from cold air, Nux-v., Puls.) Mouth always full of an unpleasant "slime;" constant hawking up of mucus. (You will recall to mind here the characteristic complaint of the Sulphur patient, that "all her troubles seem to be caused by the nauseous saliva" which fills her mouth.) We have burning in mouth and on tongue as if from blisters or from highly seasoned food.

There is a distended feeling in the stomach; a sense of weight in stomach; a full feeling extending into chest with difficult breathing; a beating in stomach with nausea; almost constant nausea; vomiting of bitter, sour slime.

Aching, sometimes cutting pains in region of liver, with great distress there. Cannot bear tight clothing around the waist (Lach., Spong., Calc., Graph., etc.); a weight in region of liver; a sensitiveness to touch, pressure, deep breathing, walking; must lie on back, pain when moving to either side. There is, too, a griping in abdomen better from kneading it. Diarrhœa, worse in wet weather and in cool, evening air (also Merc.). In cases of chronic diarrhœa where the stool is watery, is expelled with a gush accompanied with much flatus; is worse on first moving about after rising in morning.

Breathing, great dyspnœa, desires to breathe deeply during damp weather. After sunset an oppressed feeling in chest and feeling as of a ball in throat, with a hysteric tendency to cry. Asthma in young people at every change of the weather. (See China.) Empty feeling in chest with a cough that compels one to hold the chest; at night must sit up and hold the chest. (Arn., Bry., Dros., Natr-m., Sepia, Phos., all have this holding of chest on coughing.) The expectoration is thick, green, and comes up freely. When the chest rattles with mucus, with expectoration of large quantities of white mucus, with asthmatic breathing, this drug should be studied.

In cases of chronic gonorrhœa, with greenish discharge, this drug is recommended. The discharge, instead of running off into a white, gleety mucus, continues thick, yellow, and green. Natr-sul. here competes with Merc. and Thuja, both anti-sycotics; with Natr-sul. there is generally very little pain, it is almost painless.

Dr. Kent sends me a note saying that Natr-sul.$^{5c}$ produced a whitish discharge from the urethra, lasting four days, without any irritation. Also, in another case, the same potency brought back a gonorrhœal discharge many years suppressed.

This is a remedy for panaritium when the pain is easier out-of-doors; the patient is pale, thin, sickly looking; is weary, has dullness of head; is worse in mornings; especially when caused by living in damp places. On the skin we have eczema, moist and oozing profusely; sycotic, wart-like eruptions about anus and elsewhere on skin.

This remedy has a very marked aggravation in damp weather and from living in damp places; also complaints from eating fish and water plants. E. J. L.

